# Machine Learning and Deep Learning for Diagnosis of Lumbar Spinal Stenosis: Systematic Review and Meta-Analysis

**DOI:** 10.2196/54676

**Published:** 2024-12-23

**Authors:** Tianyi Wang, Ruiyuan Chen, Ning Fan, Lei Zang, Shuo Yuan, Peng Du, Qichao Wu, Aobo Wang, Jian Li, Xiaochuan Kong, Wenyi Zhu

**Affiliations:** 1 Beijing Chaoyang Hospital Capital Medical University Beijing China

**Keywords:** lumbar spinal stenosis, LSS, machine learning, ML, deep learning, artificial intelligence, AI, diagnosis, spine stenosis, lumbar, predictive model, early detection, diagnostic, older adult

## Abstract

**Background:**

Lumbar spinal stenosis (LSS) is a major cause of pain and disability in older individuals worldwide. Although increasing studies of traditional machine learning (TML) and deep learning (DL) were conducted in the field of diagnosing LSS and gained prominent results, the performance of these models has not been analyzed systematically.

**Objective:**

This systematic review and meta-analysis aimed to pool the results and evaluate the heterogeneity of the current studies in using TML or DL models to diagnose LSS, thereby providing more comprehensive information for further clinical application.

**Methods:**

This review was performed under the PRISMA (Preferred Reporting Items for Systematic Reviews and Meta-Analyses) guidelines using articles extracted from PubMed, Embase databases, and Cochrane Library databases. Studies that evaluated DL or TML algorithms assessment value on diagnosing LSS were included, while those with duplicated or unavailable data were excluded. Quality Assessment of Diagnostic Accuracy Studies 2 was used to estimate the risk of bias in each study. The MIDAS module and the METAPROP module of Stata (StataCorp) were used for data synthesis and statistical analyses.

**Results:**

A total of 12 studies with 15,044 patients reported the assessment value of TML or DL models for diagnosing LSS. The risk of bias assessment yielded 4 studies with high risk of bias, 3 with unclear risk of bias, and 5 with completely low risk of bias. The pooled sensitivity and specificity were 0.84 (95% CI: 0.82-0.86; *I*^2^=99.06%) and 0.87 (95% CI 0.84-0.90; *I*^2^=98.7%), respectively. The diagnostic odds ratio was 36 (95% CI 26-49), the positive likelihood ratio (LR+) was 6.6 (95% CI 5.1-8.4), and the negative likelihood ratio (LR–) was 0.18 (95% CI 0.16-0.21). The summary receiver operating characteristic curves, the area under the curve of TML or DL models for diagnosing LSS of 0.92 (95% CI 0.89-0.94), indicating a high diagnostic value.

**Conclusions:**

This systematic review and meta-analysis emphasize that despite the generally satisfactory diagnostic performance of artificial intelligence systems in the experimental stage for the diagnosis of LSS, none of them is reliable and practical enough to apply in real clinical practice. Further efforts, including optimization of model balance, widely accepted objective reference standards, multimodal strategy, large dataset for training and testing, external validation, and sufficient and scientific report, should be made to bridge the distance between current TML or DL models and real-life clinical applications in future studies.

**Trial Registration:**

PROSPERO CRD42024566535; https://tinyurl.com/msx59x8k

## Introduction

Lumbar spinal stenosis (LSS) is a major cause of pain and disability in older individuals [1]. LSS has become a worldwide public health issue as it is estimated that more than 102 million people are diagnosed with LSS annually, with high incidence in Europe and the United States of America [2,3]. According to the clinical guideline developed by the North American Spine Society, LSS is characterized as a condition of diminished space available for the neural and vascular elements in the lumbar spine, secondary to degenerative changes in the spinal canal [4]. An accurate LSS diagnosis is essential for treatment options and effectiveness. Currently, clinicians diagnose LSS based on a comprehensive evaluation combined with the patient’s history, physical examination, and spinal imaging tests such as x-ray, computed tomography (CT), and magnetic resonance imaging (MRI) [1,2]. As a superior radiographic screening tool for soft tissues, MRI plays a crucial role in detecting the presence, classification, and grading of LSS [5-7]. However, detailing numerous information in spinal MRI is time-consuming and repetitive, which causes laborious clinical workloads [7]. Furthermore, existing LSS grading systems are mainly qualitative or semiquantitative, which highly depend on expertise and suffer from high interobserver variations because of the complexity of the spinal canal and foramen [5,6,8-11]. Therefore, more intelligent radiographic diagnostic and grading methods of LSS are warranted.

Machine learning (ML), a subdiscipline of artificial intelligence (AI), has shown great advantages in analyzing medical imaging and predicting outcome decisions [12-14]. ML begins with algorithms trained with a set of data, such as image features, to establish the prediction or diagnosis by extracting and classifying relevant information. More recently, a crucial branch of ML, named deep learning (DL), was standing out rapidly. DL algorithms were designed with multiple processing layers, which can learn more complex image features than traditional ML methods [15]. Although DL is still challenged by the demand for large-scale datasets and the difficulty of interpretation, it owns the incomparable advantage of automatic feature extraction, minimizing the bias by manual intervention [12,14]. In 2016, He et al [16] attempted to use traditional ML (TML) methods based on their newly proposed synchronized superpixel representation model to recognize the presence of radiographic lumbar foraminal stenosis (LFS). Subsequently, increasing studies of TML and DL were conducted in the field of diagnosing and grading LSS and gained prominent results [16-34]. However, most of these studies focus either on algorithm development or clinical validation, causing great variations in experimental settings and incompleteness of evaluation parameters of accuracy and reliability. Hence, a systematic review and meta-analysis were believed to be necessary to evaluate the heterogeneity and provide comprehensive results from these studies. However, to our knowledge, no systematic review and meta-analysis was previously conducted to address this issue.

Therefore, this systematic review and meta-analysis aimed to evaluate the heterogeneity and pool the results of the current studies in using ML or DL models to diagnose LSS, thereby providing more comprehensive information for further clinical application.

## Methods

### Study Design and Registration

This systematic literature review was conducted following the PRISMA (Preferred Reporting Items for Systematic Reviews and Meta-Analyses) guidelines and flowchart [35,36] and the PRISMA diagnostic test accuracy checklist (Multimedia Appendix 1) [37]. The protocol for this systematic review was registered in PROSPERO (ID: CRD42024566535). Ethical approval was not required because this systematic literature review focused on retrospective studies.

### Search Strategy

This review collected the records from 3 major databases up to October 2023. A second search was performed in February 2024 to complement newly published studies. Those databases include PubMed, Embase, and the Cochrane Library (CENTRAL), which are recommended academic search systems for systematic reviews and meta-analyses [38]. We used the MeSH (Medical Subject Headings) and Emtree headings in several combinations and supplemented them with free text to increase sensitivity. In addition, we searched references contained in the included studies to supplement the relevant literature. An experienced librarian designed and implemented the search strategy. The following MeSH terms were used for PubMed: “Spinal Stenosis,” “Intervertebral Disc Degeneration,” “Lumbar Vertebrae,” “Machine Learning,” “Deep Learning,” and “Neural Networks, Computer*.” The details of the search strategy are stated in Multimedia Appendix 2.

### Inclusion and Exclusion Criteria

We included studies that evaluated the assessment value of DL or TML algorithms for diagnosing LSS and that were available in English. The included studies in the meta-analysis should provide or could be reconstructed as a 2×2 confusion matrix from sensitivity, specificity, and precision. Applied statistical, non–artificial intelligence, and general AI methods are not considered DL or TML. Articles with duplicated or unavailable data were excluded. Furthermore, abstracts from protocols, case reports, editorials, and review articles were excluded.

### Review Process

A total of 2 reviewers (TW and NF) independently performed an initial screening of the titles and abstracts of the remaining articles to determine potential eligibility after removing duplicates. We reviewed the full texts of the remaining articles and excluded those that did not meet the inclusion criteria. We searched and screened a list of references for all relevant studies and a systematic review of potentially relevant studies. Disagreements were resolved by discussion and by third-party adjudication when necessary. For studies enrolled in systematic review while lack of available data for meta-analysis, an email was sent to the corresponding authors for acquisition of the necessary data.

### Data Extraction

A total of 2 reviewers independently extracted, summarized, and tabulated the following data using a standard form: baseline characteristics of studies, including the publication year, study type, model type, algorithms used, LSS classifications, number of participants, validation strategy, imaging modality, and diagnosis criteria of LSS. Any discrepancies in the extracted data were resolved by discussion. For the studies that provided multiple contingency tables based on different classifier algorithms, datasets, LSS types, or label strategies, we assumed these to be independent of each other. For the studies that provided multiple contingency tables based on different preprocessing strategies, we selected the best-performing result. If there was no preprocessing strategy that performed significantly better than the others, we also enrolled each strategy as an individual study and collected the corresponding results. For repeat test results based on the same classifier algorithms, datasets, and so on, we calculated the average values of metrics as the final results.

### Quality Assessment

A total of 2 reviewers (TW and NF) used the Quality Assessment of Diagnostic Accuracy Studies 2 (QUADAS-2), which is a tool for assessing the quality of primary diagnostic accuracy studies, to independently assess the risk of bias for each eligible study [39]. The QUADAS-2 criteria assessed the risk of bias in 4 domains: patient selection, index test, reference standard, and flow and timing. Any disagreements were resolved by discussion with a third author.

### Statistical Analysis

We used the MIDAS module and the METAPROP module [40] of Stata (version 17.0; StataCorp) for statistical analysis. Postestimation procedures for model diagnostic were used to assess heterogeneity using the *I*^2^ statistic. The following metrics were used: 0%-40% (low heterogeneity), 30%-60% (moderate heterogeneity), 50%-90% (substantial heterogeneity), and 75%-100% (considerable heterogeneity). Bivariate mixed-effects logistic regression modeling was conducted, and forest plots were used to compare the sensitivity and specificity of DL or TML models for diagnosing LSS. We used summary receiver operating characteristic (SROC) curves to assess overall diagnostic accuracy. We used the Fagan nomogram to explore the relationship between pretest probability, likelihood ratio (LR), and posttest probability. LR dot plots were divided into 4 quadrants according to the strength of the evidence threshold, which was used to determine DL or TML model exclusion and confirmation. Finally, subgroup analyses were performed to examine whether the estimated sensitivity, specificity, and associated *I*^2^ differed by several moderators when each subgroup included ≥4 datasets.

## Results

### Study Selection and Characteristics

**Figure 1:**
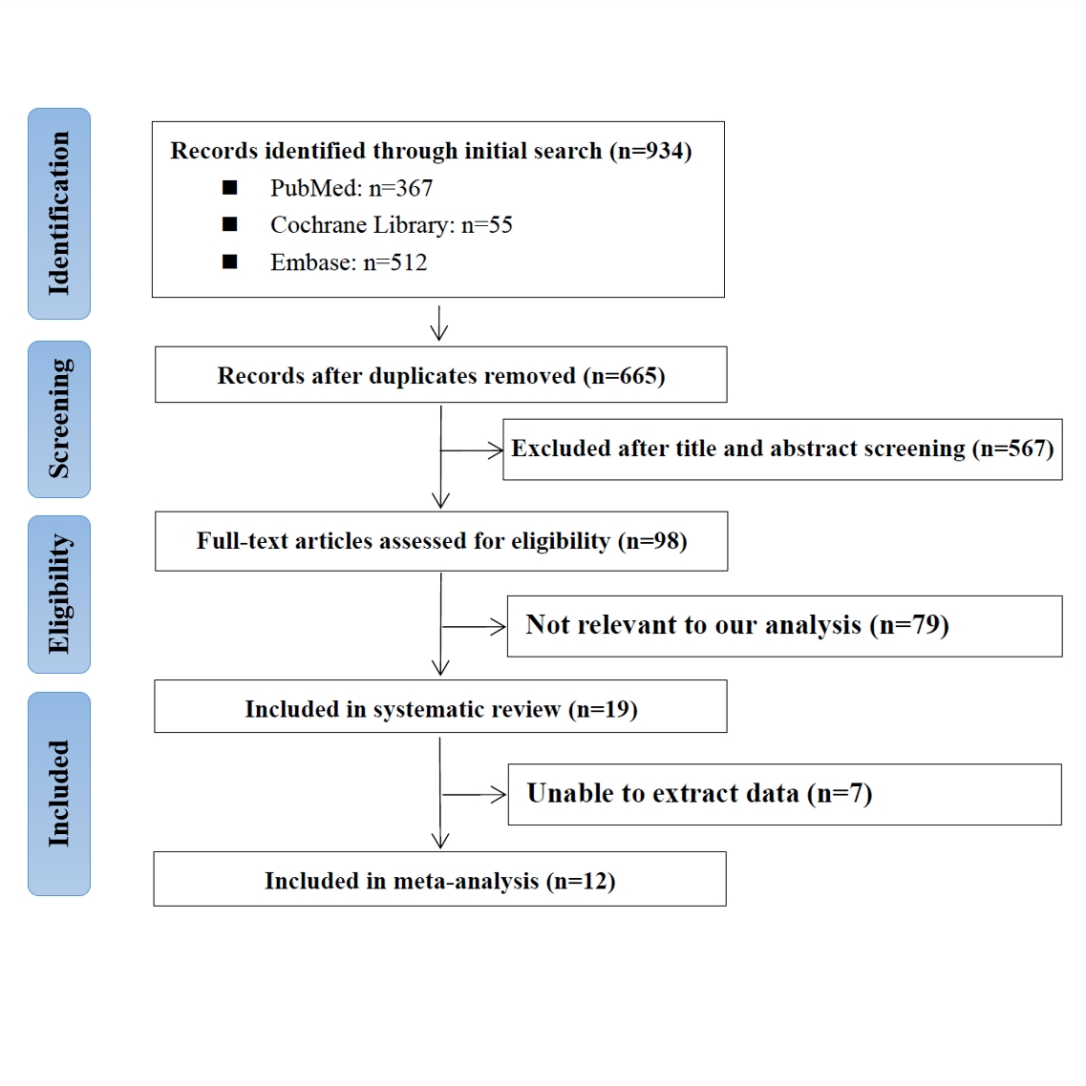
Flowchart depicting PRISMA (Preferred Reporting Items for Systematic Reviews and Meta-Analyses) search strategy.

The initial search identified 934 titles and abstracts, of which 269 were duplicates. After screening, 567 articles were excluded following this study’s inclusion and exclusion criteria. In addition, 98 studies were reviewed for full text, of which 19 and 12 studies were included in the systematic review and meta-analysis, respectively (Figure 1). Table 1 summarizes the characteristics of the studies in the systematic review and meta-analyses, including study type, model type, algorithms used, LSS classifications, number of participants, validation strategy, imaging modality, and diagnosis criteria of LSS. The 19 studies included in the systematic review were published from 2016 to 2024. The 12 studies included in the meta-analysis were all retrospective and included 21 external tests [17,25-27,30,32,34] and 35 internal tests [17,20,22,24,26-28,33,34]. Therefore, the meta-analysis included 56 datasets and completely different data sources. Among the 56 datasets, 32 identified LSS on MRI [17,20,22,24,25,27,28,30,32], 20 on x-ray [26,34], and 4 on CT [33]. Furthermore, 29 datasets have developed and internally tested DL models [17,20,24,26-28,33,34], 6 datasets internally tested TML models [22,28], and 21 datasets externally tested the DL models [17,25-27,30,32,34].

**Table 1:** Characteristics of the included studies in the systemic review and meta-analysis.

Study	Study type	Model type	Algorithms used^a^	LSS^b^ type	Number of participants, n	Validation strategy	Imaging modality	Diagnosis criteria
He et al [16]	Development study and internal test	TML^c^	KNN^d^ and SVM^e^ and LDA^f^	LFS^g^	110	Cross-validation	MRI^h^	Lee et al [5]
Jamaludin et al [18]	Development study and internal test	DL^i^	CNN^j^ (SpineNet^k^)	LCS^l^	2009	Hold-out validation	MRI	No reference
Zhang et al [19]	Development study and internal test	TML	SVM and Decision Tree	LCS and LFS	600	Hold-out validation	MRI	No reference
Lu et al [21]	Development study and internal test	DL	ResNeXt-50	LCS and LFS	4075	Hold-out validation	MRI	No reference
Han et al [20]^m^	Development study and internal test	DL	CNN and FCN and SegNet and DeepLabv3+ and U-Net	LFS	253	Cross-validation	MRI	Lee et al [5]
Huber et al [22]^m^	Development study and internal test	TML	Decision Tree	LCS	82	Cross-validation	MRI	Lee et al [9] and Schizas et al [6]
Ishimoto et al [23]	Replication study and internal test	DL	CNN (SpineNet^k^)	LCS	971	Hold-out validation	MRI	Lurie et al [8]
Won et al [24]^m^	Development study and internal test	DL	VGG	LCS	542	Cross-validation	MRI	Schizas et al [5]
Hallinan et al [17]^m^	Development study and internal test and external test	DL	CNN	LCS and LRS^n^ and LFS	446/100	Hold-out validation	MRI	Lurie et al [8] and Bartynski and Lin [10]
Lehnen et al [25]^m^	External test	DL	CNN (CoLumbo^k^)	LCS	146	—^o^	MRI	Lee et al [9]
Grob et al [30]^m^	External test	DL	CNN (SpineNet^k^)	LCS	882	—	MRI	Lurie et al [8]
Kim et al [26]^m^	Development study and internal test and external test	DL	VGG19 and VGG16 and ResNet50 and Efficient1	LCS	4644/199	Cross-validation	X-Ray	Lee et al [9]
Su et al [27]^m^	Development study and internal test and external test	DL	ResNet-50	LCS	1015/100	Hold-out validation	MRI	Lee et al [9] and Park et al [11]
Altun et al [28]^m^	Development study and internal test	TML and DL	RF and SVM and VGG16 and ResNet and MobileNet and InceptionNet	LSS	1030	Cross-validation	MRI	No reference
Bharadwaj et al [29]	Development study and internal test	TML and DL	Decision Tree and BiTCNN	LCS and LFS	200	Hold-out validation	MRI	Schizas et al [6] and Lee et al [5]
Tumko et al [32]^m^	Development study and external test	DL	RegNetY32GF	LCS and LRS and LFS	1635/150	—	MRI	Schizas et al [6]
Shahzadi et al [31]	Development study and internal test	DL	CNN	LRS and LFS	515	Cross-validation	MRI	No reference
Li et al [33]^m^	Development study and internal test	DL	VGG11 and ResNet-18	LCS and LRS	236	Hold-out validation	CT	Lurie et al [8] and Bartynski and Lin [10]
Park et al [34]^a^	Development study and internal test and extra-internal test and external test	DL	ResNet50 and VGG19 and VGG16 and EfficientNet-B1	LCS	3831/199/100	Cross-validation (validation) and Hold-out validation (Internal test)	X-Ray	No reference

^a^Algorithms for only classifiers.

^b^LSS: lumbar spinal stenosis.

^c^TML: traditional machine learning.

^d^KNN: k-nearest neighbors.

^e^SVM: support vector machine.

^f^LDA: linear discriminant analysis.

^g^LFS: lumbar foraminal stenosis.

^h^MRI: magnetic resonance imaging.

^i^DL: deep learning.

^j^CNN: convolutional neural network.

^k^Name of software.

^l^LCS: lumbar central stenosis.

^m^Studies included in meta-analysis (confusion matrix available or can be reconstructed).

^n^LRS: lateral recess stenosis.

^o^Not applicable.

### Methodological Quality

**Figure 2:**
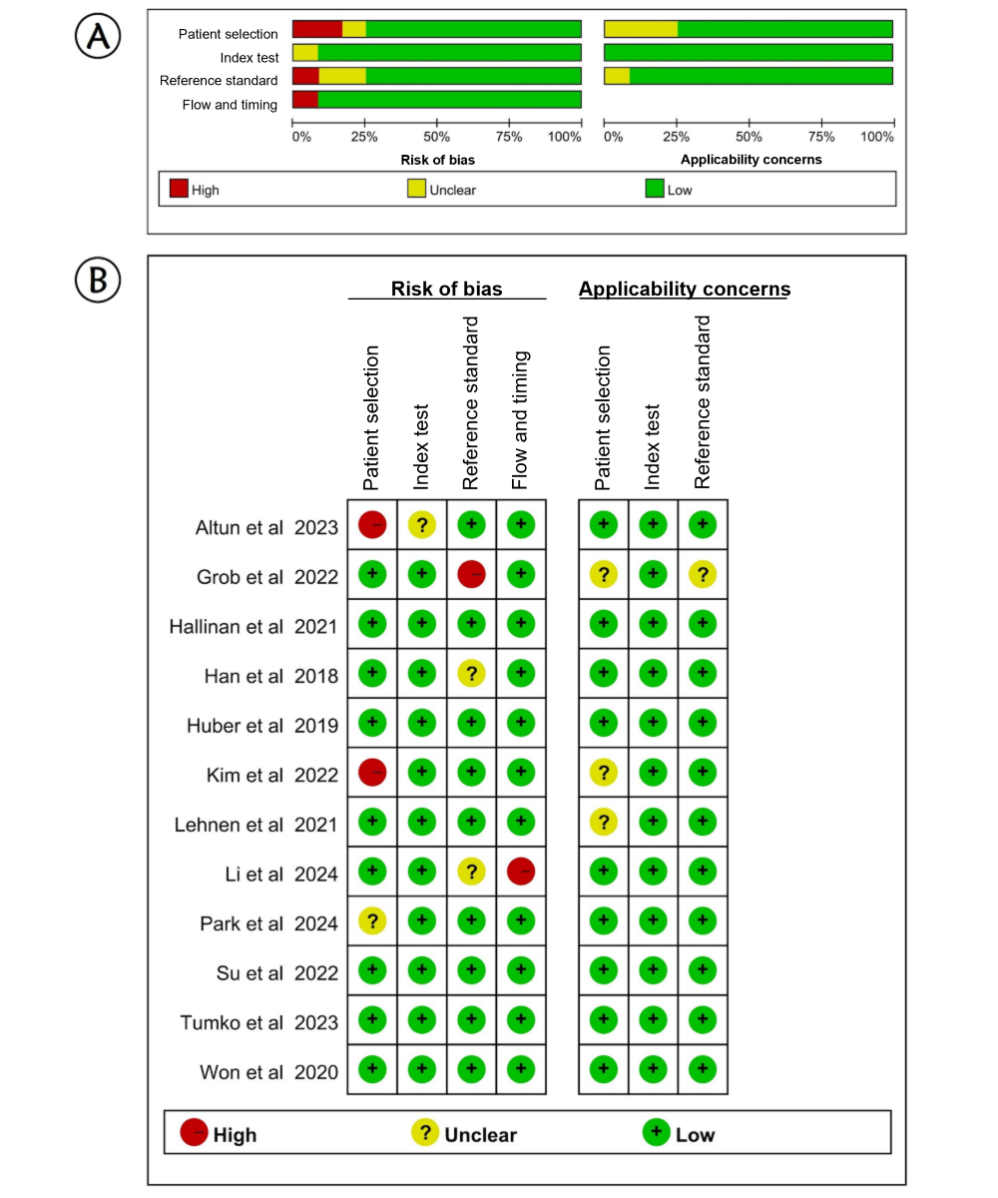
Methodological assessment by Quality Assessment of Diagnostic Accuracy Studies-2 (QUADAS-2). (A) The proportion of risk of bias for all domains and proportion of applicability concerns in three domains. (B) Summary of the risk of bias for each study. Green, yellow, and red circles indicate low, unclear, and high risk of bias, respectively [8,20,22,24-28,30,32-34].

Regarding the QUADAS-2 risk of bias assessment (Figure 2 [17,20,22,24-28,30,32-34]), we revealed 4 studies with a high risk of bias [26,28,30,33], 3 with an unclear risk of bias [20,25,34], and 5 with a completely low risk of bias [17,22,24,27,32]. In particular, 2 of the included studies reported no details of patient selection [26,28], causing a high bias in patient selection. Furthermore, 1 study provided unclear information on how to perform the index test [28], thereby causing an unclear risk of bias. Furthermore, 1 study used the improper reference standard, which was not likely to correctly classify the target condition [30], causing a high risk of bias in the reference standard. Besides, 1 study showed a high risk of bias with regard to flow and timing issues [33].

### Performance of TML and DL Models for LSS

**Figure 3:**
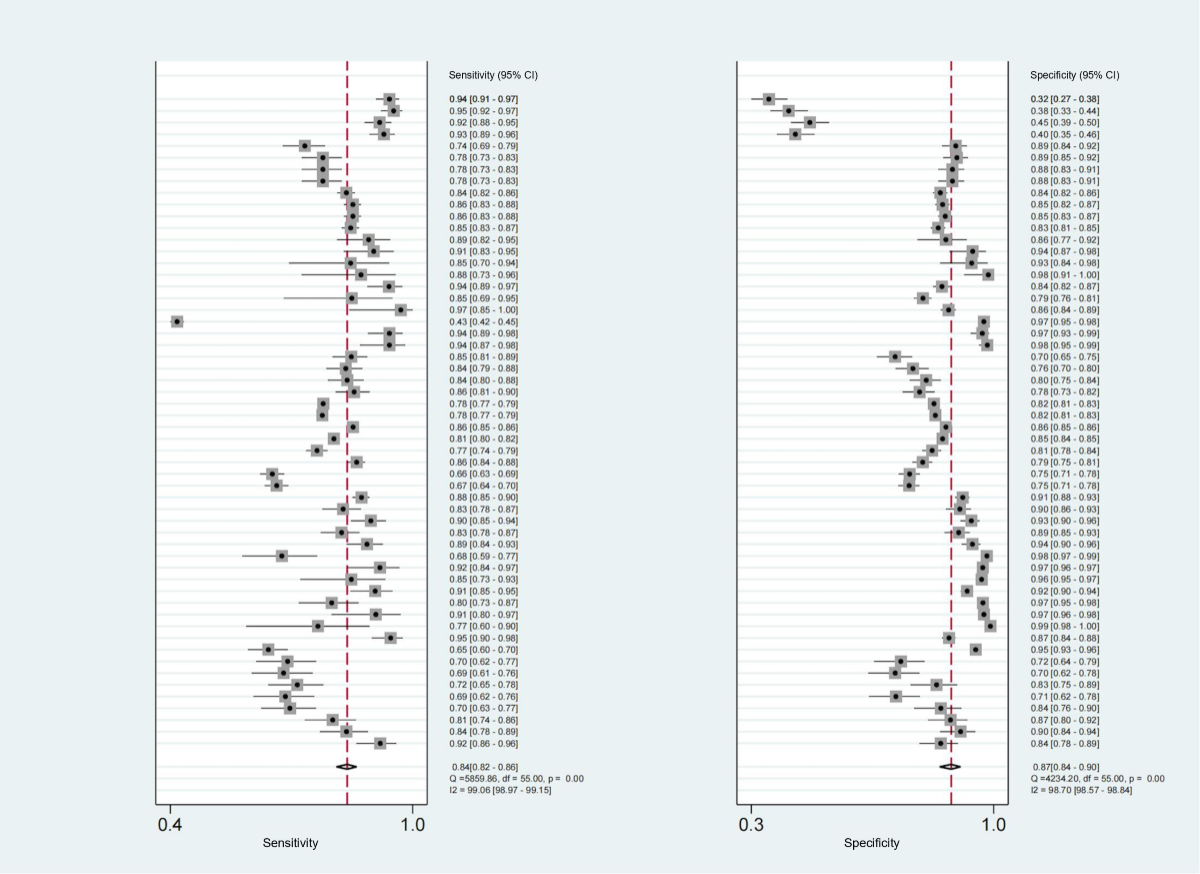
Forest plots in sensitivity and specificity of traditional machine learning (TML) or deep learning (DL) models. The pooled sensitivity and specificity were 0.84 (95% CI 0.82-0.86) and 0.87 (95% CI 0.84-0.90), respectively.

**Figure 4:**
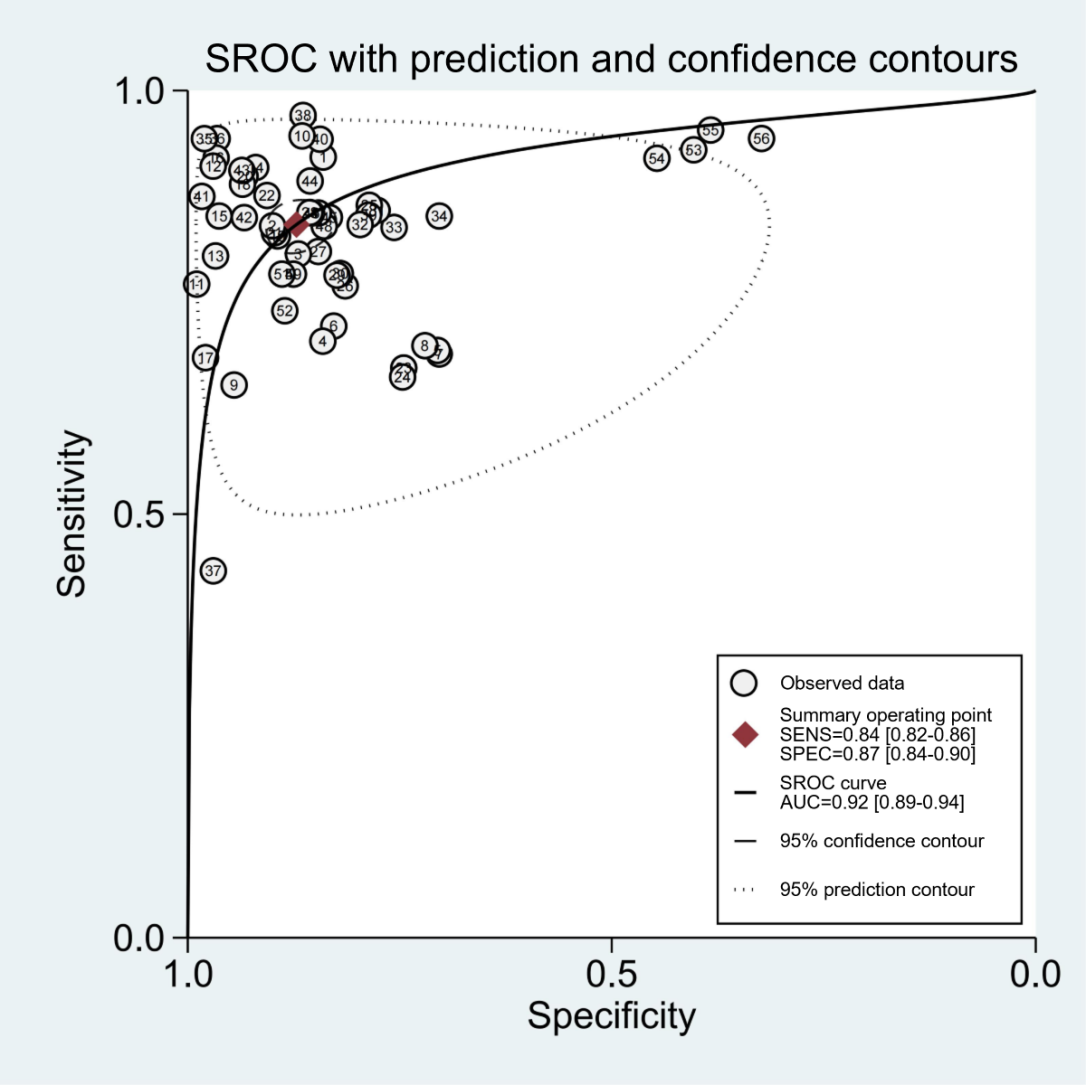
The SROC graph for the studies. The AUC of summary receiver operating characteristic (TML) or deep learning (DL) models for diagnosing LSS was 0.92 (95% CI 0.89-0.94). SROC: summary receiver operating characteristic; AUC: area under the curve; SENS: sensitivity; SPEC: specificity.

A total of 12 studies with 15,044 patients reported the assessment value of TML or DL models for diagnosing LSS. The pooled sensitivity was 0.84 (95% CI 0.82-0.86; *I*^2^=99.06%), and specificity was 0.87 (95% CI 0.84-0.90; *I*^2^=98.7%; Figure 3). The diagnostic odds ratio was 36 (95% CI 26-49). The SROC curve (Figure 4) revealed that the area under the curve of TML or DL models for diagnosing LSS was 0.92 (95% CI 0.89-0.94), indicating a high diagnostic value.

**Figure 5:**
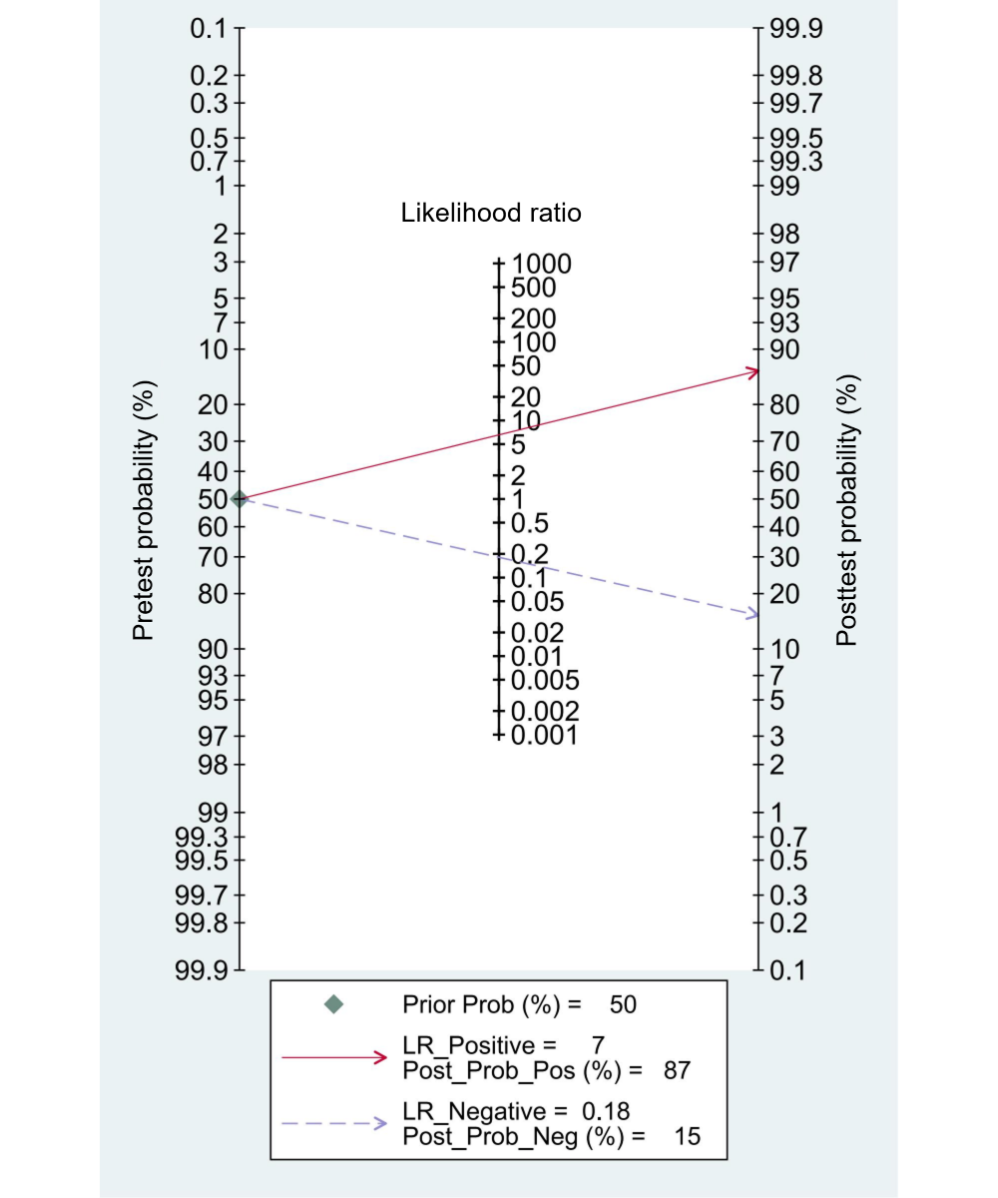
Fagan nomogram of traditional machine learning (TML) or deep learning (DL) models for diagnosing lumbar spinal stenosis (LSS). The first column of this nomogram represents the pretest probability, the second column represents the likelihood ratio, and the third shows the posttest probability.

**Figure 6:**
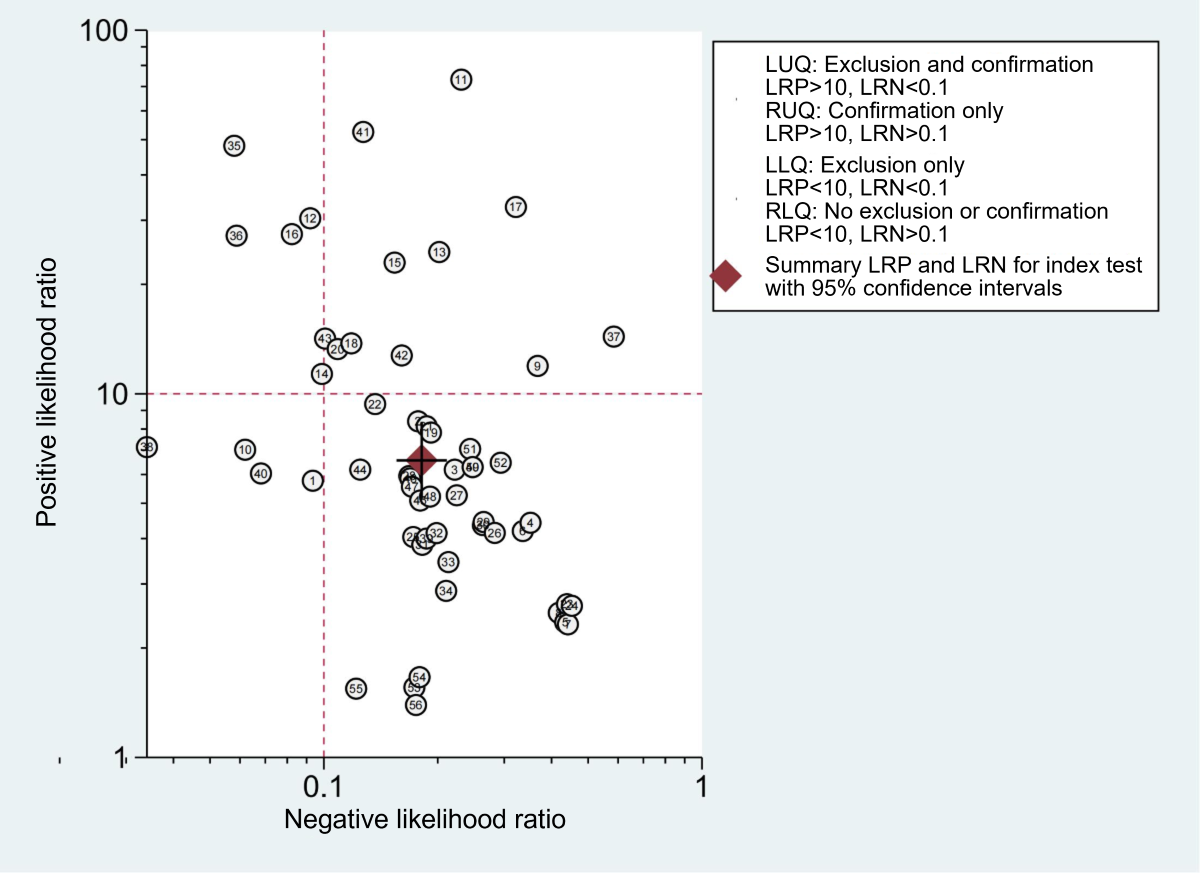
Likelihood ratio (LR) dot plot of traditional machine learning (TML) or deep learning (DL) prediction models. The summary point of TML or DL models was in the right lower quadrant (LR+<10 and LR–>0.1: no exclusion or confirmation). LRN: negative likelihood ratio; LRP: positive likelihood ratio; LUQ: left upper quadrant; RLQ: right lower quadrant; RUQ: right upper quadrant.

We set the pretest probability to 50% based on the pretest probability of disease. At this point, true positives accounted for 87% when patients were diagnosed with LSS by the TML or DL model, and false negatives accounted for 15% when the diagnosis was not LSS (Figure 5). Furthermore, the models showed a positive likelihood ratio (LR+) of 6.6 (95% CI 5.1-8.4) and a negative likelihood ratio (LR–) of 0.18 (95% CI 0.16-0.21), respectively (Figure 5). However, the summary likelihood ratio plot of TML or DL models was in the right lower quadrant (LR+<10 and LR–>0.1: no exclusion or confirmation), and the individual plots were scattered and distributed (Figure 6). The results indicated that although the TML or DL models achieved an acceptable performance generally, it was still insufficient enough for diagnosing or excluding LSS, and the current models suffered from certain performance variations.

In total, 4 studies [17,23,29,32] simultaneously provided the performance of reliability both of observers and TML or DL models, including 3 studies [17,29,32] that performed a direct comparison between their reliabilities based on the same assessment datasets (Table 2).

**Table 2:** Characteristics of the studies available for the agreement between models and observers and reference standard.

Study	Number of participants, n	Agreement assessment strategy	Control group	Model type	Type of classification		LSS^a^ type	Model results	Control group results
Hallinan et al [17]	446	Gwet ĸ	2 radiologists	DL^b^		Binary	LCS^c^	0.96	0.98/0.98
Hallinan et al [17]	446	Gwet ĸ	2 radiologists	DL		Binary	LRS^d^	0.92	0.92/0.95
Hallinan et al [17]	446	Gwet ĸ	2 radiologists	DL		Binary	LFS^e^	0.89	0.94/0.95
Hallinan et al [17]	446	Gwet ĸ	2 radiologists	DL		Multigrading	LCS	0.82	0.89/0.89
Hallinan et al [17]	446	Gwet ĸ	2 radiologists	DL		Multigrading	LRS	0.72	0.71/0.79
Hallinan et al [17]	446	Gwet ĸ	2 radiologists	DL		Multigrading	LFS	0.75	0.80/0.87
Bharadwaj et al [29]	200	Cohen ĸ	2 radiologists	DL	Multigrading		LCS	0.54	0.80/0.86
Bharadwaj et al [29]	200	Cohen ĸ	2 radiologists	TML^f^	Multigrading		LCS	0.80	0.80/0.86
Tumko et al [32]	150	Cohen ĸ	7 radiologists	DL	Binary		LCS	0.431	Average 0.372
Tumko et al [32]	150	Cohen ĸ	7 radiologists	DL	Binary		LRS	0.315	Average 0.323
Tumko et al [32]	150	Cohen ĸ	7 radiologists	DL	Binary		LFS	0.672	Average 0.596
Tumko et al [32]	150	Cohen ĸ	7 radiologists	DL	Multigrading		LCS	0.310	Average 0.376
Tumko et al [32]	150	Cohen ĸ	7 radiologists	DL	Multigrading		LRS	0.199	Average 0.359
Tumko et al [32]	150	Cohen ĸ	7 radiologists	DL	Multigrading		LFS	0.637	Average 0.620

^a^LSS: lumbar spinal stenosis.

^b^DL: deep learning.

^c^LCS: lumbar central stenosis.

^d^LRS: lateral recess stenosis.

^e^LFS: lumbar foraminal stenosis.

^f^TML: traditional machine learning.

### Subgroup Analysis

We conducted the subgroup analyses in 3 areas, including data partition (internal test or external test), model networks (TML or DL), and image (MRI or x-ray), to effectively understand how the 3 different types affected the performance of the algorithm for LSS assessment (Table 3). The internal test group demonstrated a lower sensitivity (*P*<.01) yet higher specificity (*P*<.01) than the external test group. Besides, the MRI group showed a lower sensitivity (*P*<.01) yet higher specificity (*P*<.01) than the x-ray group. The sensitivity in the DL group achieved 0.85, which was significantly higher than that (0.80) in the TML group (*P*<.01). Meanwhile, the DL group showed a more stable performance on specificity than the TML group (*P*=.04).

**Table 3:** Results of subgroup analysis.

Categories		Studies, n	Sensitivity (95% CI^a^)	*P* value (HBG^b^ of sensitivity)		Specificity (95% CI)		*P* value (HBG of specificity)	
**Data partition**					<.001				<.001
	Internal test	35	0.83 (0.80-0.86)			0.89 (0.85-0.92)			
	External test	21	0.86 (0.82-0.90)			0.85 (0.79-0.91)			
**Model networks**					<.001				.04
	TML^c^	6	0.80 (0.72-0.89)			0.87 (0.77-0.97)			
	DL^d^	50	0.85 (0.82-0.87)			0.87 (0.84-0.91)			
**Image**					<.001				<.001
	MRI	32	0.83 (0.79-0.86)			0.91 (0.88-0.93)			
	X-ray	20	0.85 (0.82-0.89)			0.77 (0.70-0.84)			

^a^CI: confidence interval.

^b^HBG: heterogeneity between groups.

^c^TML: traditional machine learning.

^d^DL: deep learning.

## Discussion

### Principal Findings

In recent years, there has been a boom in assessing the diagnosis and grading of LSS by TML or DL methods. After systemically reviewing the available evidence, we revealed that all related studies were published after 2016 and increased annually. It can also be said that TML and DL algorithms have been showing promising potential in this field. To the best of our knowledge, this is the first systematic review and meta-analysis for addressing this issue. Our pooled results showed an overall sensitivity of 0.84 and a specificity of 0.87 for diagnosing LSS by TML or DL models. The area under the SROC was 0.92, indicating a high diagnostic value. Subgroup analysis revealed a better diagnostic performance in internal validation than in external validation, while DL algorithms demonstrated higher sensitivity and specificity than TML algorithms. However, 37% of studies enrolled in the systemic review were unavailable in the meta-analysis, which may have caused a discrepancy between pooled results and reality. Therefore, the results should be interpreted with caution.

A permanent debate focuses on whether the diagnostic performance of ML or DL algorithms surpassed that of clinicians. High-level evidence showed that the performance of AI diagnostic systems is equivalent to health care professionals, and AI-assistance systems improve clinician diagnostic performance [41-43]. However, in the field of LSS, there were few studies designed to directly compare the performance of additional radiologists or orthopedic surgeons with ML or DL algorithms in the same dataset. Hallinan et al [17] developed a DL method for diagnosing different LSS and compared the sensitivity and specificity of the DL model with 2 independent clinicians (a neuroradiologist and a musculoskeletal radiologist) with less than 10 years of experience. The study revealed that the sensitivity of DL in detecting LSS was on par with clinicians in general, with even slightly higher in lumbar central stenosis (LCS) and lateral recess stenosis (LRS), but with lower specificity of DL. It is reasonable because pursuing sensitivity to reduce false-negative results on the premise of maximizing the accuracy and AUC may be an alternative and beneficial method for clinical demands [44]. Compared with the complete replacement of clinicians, AI diagnostic systems are more expected to be assisted screening tools to use in areas with poor medical resources without experts or to reduce the workload of clinicians and missed diagnoses, followed by high-level medical team screening of image marked positive by the automatic diagnosis [45].

Although the general performance of diagnostic models was satisfactory, it was still insufficient enough for diagnosing or excluding LSS according to the summary likelihood ratio plot. Besides, our systemic review and meta-analysis found that ML or DL models showed similar, even slightly lower, sensitivity compared with specificity in general, especially in the MRI modality. There may be several reasons. First, the complexity and variety of pathological structures in individuals with LSS result in no broadly accepted quantitative radiologic evidence for diagnosis, even in expert evaluation [46], which makes automatic detection by MRI difficult. Furthermore, we cannot exclude that the results may be influenced by heterogeneity. Consideration should be taken for developers to optimize models prone to higher sensitivity than specificity for diagnosis and grading of LSS, which may be more beneficial to clinical workflow.

Notably, a consensus of reference standards in determining ML or DL performance for diagnosing LSS has not been reached till now. The reference standards in almost all included studies were labeled by qualitative or semiquantitative expert evaluation, which suffered from considerable heterogeneity due to the different amount, specialties, and years of experience of experts. Huber et al [22] combined texture analysis and decision trees to detect LSS based on the cross-sectional area (CSA) as a quantitative reference standard. However, a CSA of <130mm^2^ was not a widely accepted criterion, and it is only appropriate for LCS, while quantitative radiological criteria remain unavailable for diagnosing LRS or LFS [46,47]. More comprehensive and rigorous criteria for reference standards should be developed in future work. In addition, the diagnosis of LSS should combine the imaging findings with history and clinical presentation because LSS is a clinical syndrome, and solely radiographic LSS may be symptom-free [2]. However, the diagnosis criteria in all reviewed studies were only based on radiographic criteria or reports, which means that the current TML or DL models were developed for the diagnosis of radiographic LSS objectively. Yet, it is not said that radiographic evaluation is valueless for LSS. On the one hand, it can provide details in pathological anatomy, which guides further treatment options and surgical approaches. On the other hand, a potentially imperceptible relationship between radiographic characters and clinical LSS may be explored with the help of AI models. Therefore, we suggest attempting to label the data by clinical LSS as golden standards on the premise of model interpretability and eliminating confounding factors. Furthermore, developers can set multiple data types, such as crucial details of patient’s history, physical examination, and imaging tests, as inputs to build a multimodal to improve the clinical value of LSS diagnosis and grading by AI approaches [48].

Overall, our meta-analysis revealed a better performance for diagnosing LSS in DL than in TML. Whereas results should be interpreted with caution because of the limited number of enrolled studies on TML in meta-analysis. Only 2 included studies in the systemic review designed a direct comparison of the capability of DL and TML models for diagnosing LSS, yet showed contradictory results. Altun et al [28] found that VGG16 and 3 other DL techniques performed better in addressing the issue of binary LSS classification compared with random forest and support vector machines. Conversely, Bharadwaj et al [29] combined segmentation with DL and TML classifiers to conduct multiclass and binary LSS grading. Both accuracy, AUC, and reproducibility were higher in the TML group [29]. The inconsistency may be attributed to the scale of training data. DL was generally acknowledged as the most outstanding ML technique for automatic medical image analysis [49]. However, DL is restricted to a stronger data dependency compared with any other ML, as it is designed with a more complex architecture [14,15,50]. In particular, there is an extreme need for DL to be trained with a sufficiently large sample set, particularly considering the complexity of spinal MRI. In the 2 studies above, the scales of training samples were more than 5 times higher in the study of Altun et al [28] than that of Bharadwaj et al [29] (927 vs 170). Hence, we recommend that a larger scale dataset for training both TML and DL models is beneficial for exploring their capabilities in diagnosing and grading LSS in order to reduce data overfitting and improve the performance of models.

Any AI diagnostic systems should be clinically oriented instead of technically oriented. This poses a challenge for developers in developing ML or DL models more appropriately for clinical practice rather than more technically challenging. Currently, although promising results in exactitude (accuracy, sensitivity, etc) have been widely reported, other aspects of great clinical significance, like reliability, usability, and safety, were rarely assessed. A good agreement between the model and reference standard can verify the validity and reliability of the model. However, only 3 included studies performed a direct comparison of reliabilities between models and observers [17,29,32], and a generally higher consistency of diagnosis was achieved by clinicians than that of ML or DL systems. Besides, external validation is a valuable approach to validate the generalizability of ML or DL algorithms, testing their capabilities for adapting the differences between initial settings of data collection, imaging tests, and imaging processing with replication or real-world settings [45]. Inspiringly, this meta-analysis showed the performances of external validation were generally on par with that of the internal validation, with better sensitivity but worse specificity. However, the results may be inconclusive because only 37% of studies (7/19) enrolled in this systemic review tested the models by external validation (separate datasets for model validation only) [17,25-27,30,32,34].

Anyway, a gap remains between current ML or DL algorithms for diagnosing and grading LSS with real clinical applications. A recent review highlighted the importance of large-scale and mixed-source datasets, clinician collaboration, and a clear statement of data collection to facilitate DL in clinical applications [15]. Furthermore, only a few software, such as SpineNet (University of Oxford) [18,23,30] and CoLumbo (SmartSoft Ltd) [25], were introduced into public view despite several AI models having been developed in this field. We urged that the exploration of software design may be beneficial to extend the application of AI diagnostic models.

### Limitations

Several limitations exist in this systematic review and meta-analysis. First, most of the enrolled studies were conducted under small sample sizes, and only 6 studies (32%) had a sample size >1000 [18,21,26,27,32,34]. However, a large-scale dataset is warranted for both training and validation in AI diagnostic algorithms, especially for DL algorithms [14,15,50]. Second, few models performed external validation to test the reproducibility and extensibility. Thus, the reported performance should be interpreted with caution. Third, only a few studies provided a contingency table, while the incompleteness of reported performance metrics made it difficult to conduct a comprehensive meta-analysis, which a recent systematic review and meta-analysis in the spine field also mentioned [51]. This may cause a discrepancy between pooled results and reality. Finally, the risk of bias in this study was identified by the QUADAS-2, which is more suitable for traditional diagnostic models [52]. A more specific and practical guideline for diagnostic AI models remains under development [53].

### Conclusions

This systematic review and meta-analysis emphasize that despite the generally satisfactory diagnostic performance of artificial intelligence systems in the experimental stage for the diagnosis of LSS, none of them is reliable and practical enough to apply in real clinical practice. Further efforts, including optimization of model balance, widely accepted objective reference standards, multimodal strategy, large dataset for training and testing, external validation, and sufficient and scientific report, should be made to bridge the distance between current TML or DL models and real-life clinical applications in future studies.

## Appendix

Multimedia Appendix 1 PRISMA-DTA (Preferred Reporting Items for Systematic Reviews and Meta-Analyses extension for diagnostic test accuracy) checklist.

Multimedia Appendix 2 Details of search strategy.

## Abbreviations

AI: artificial intelligence
CSA: cross-sectional area
CT: computed tomography
DL: deep learning
LCS: lumbar central stenosis
LFS: lumbar foraminal stenosis
LR: likelihood ratio
LRS: lateral recess stenosis
LSS: lumbar spinal stenosis
MeSH: Medical Subject Headings
ML: machine learning
MRI: magnetic resonance imaging
PRISMA: Preferred Reporting Items for Systematic Reviews and Meta-Analyses
QUADAS-2: Quality Assessment of Diagnostic Accuracy Studies 2
SROC: summary receiver operating characteristic
TML: traditional machine learning
