# Supplementary material for: Machine Learning and Deep Learning for Diagnosis of Lumbar Spinal Stenosis: Systematic Review and Meta-Analysis
Source: J Med Internet Res. 2024 Dec 23;26:e54676. doi: 10.2196/54676 (PMC11704645; doi:10.2196/54676)
Supplement: Multimedia Appendix 2 [file jmir_v26i1e54676_app2.docx]

(((Machine Learning[Text Word]) OR (Deep Learning[Text Word])) OR (Neural Network[Text Word])) AND ((((spinal stenosis[Text Word]) OR (Intervertebral Disc Degeneration[Text Word])) OR (Lumbar Vertebrae[Text Word]))).

(((Machine Learning[MeSH Terms]) OR (Deep Learning[MeSH Terms])) OR (Neural Networks, Computer*[MeSH Terms])) AND (((spinal stenosis[MeSH Terms]) OR (Intervertebral Disc Degeneration[MeSH Terms])) OR (Lumbar Vertebrae[MeSH Terms])).

To conduct a thorough search, two reviewers independently and manually examined previous reviews and the reference lists of pertinent studies to identify eligible studies.
